# *Clonorchis sinensis*-Derived Protein Attenuates Inflammation and New Bone Formation in Ankylosing Spondylitis

**DOI:** 10.3389/fimmu.2021.615369

**Published:** 2021-02-25

**Authors:** Yu Jeong Lee, Moon-Ju Kim, Sungsin Jo, So-Hee Jin, Pu-Reum Park, Kijeong Park, Ho-Chun Song, Jahae Kim, Ji-Young Kim, Seung Cheol Shim, Tae-Hwan Kim, Sung-Jong Hong, Hyundeok Kang, Tae-Jong Kim, Eun Jeong Won

**Affiliations:** ^1^ Department of Parasitology and Tropical Medicine, Chonnam National University Medical School, Gwangju, South Korea; ^2^ Department of Rheumatology, Hanyang University Institute for Rheumatology Research, Seoul, South Korea; ^3^ Department of Rheumatology, Chonnam National University Medical School and Hospital, Gwangju, South Korea; ^4^ Department of Nuclear Medicine, Chonnam National University Medical School and Hospital, Gwangju, South Korea; ^5^ Division of Rheumatology, Daejeon Rheumatoid & Degenerative Arthritis Center, Chungnam National University Hospital, Daejeon, South Korea; ^6^ Department of Rheumatology, Hanyang University Hospital for Rheumatic Diseases, Seoul, South Korea; ^7^ Department of Medical Environmental Biology, Chung-Ang University College of Medicine, Seoul, South Korea; ^8^ Department of Biomedical Systems Informatics, Brain Korea 21 PLUS Project for Medical Science, Yonsei University College of Medicine, Seoul, South Korea

**Keywords:** ankylosing spondylitis, *Clonorchis sinensis*, inflammation, new bone formation, parasite

## Abstract

Helminth infections and their components have been shown to have the potential to modulate and attenuate immune responses. The objective of this study was to evaluate the potential protective effects of *Clonorchis sinensis*-derived protein (CSp) on ankylosing spondylitis (AS). Cytotoxicity of CSp at different doses was assessed by MTS and flow cytometry before performing experiments. Peripheral blood mononuclear cells (PBMCs) and synovial fluid mononuclear cells (SFMCs) were obtained from AS patients. Inflammatory cytokine-producing cells were analyzed using flow cytometry. The levels of INF-**γ**, IL-17A, TNF-α, and IL-6 were measured by enzyme-linked immunosorbent assay (ELISA). SKG mice were treated with CSp or vehicles. Inflammation and new bone formation were evaluated using immunohistochemistry, positron emission tomography (PET), and micro-computed tomography (CT). Treatment with CSp resulted in no reduced cell viability of PBMCs or SFMCs until 24 h. In experiments culturing PBMCs and SFMCs, the frequencies of IFN-**γ** and IL-17A producing cells were significantly reduced after CSp treatment. In the SKG mouse model, CSp treatment significantly suppressed arthritis, enthesitis, and enteritis. Micro-CT analysis of hind paw revealed reduced new bone formation in CSp-treated mice than in vehicle-treated mice. We provide the first evidence demonstrating that CSp can ameliorate clinical signs and cytokine derangements in AS. In addition, such CSp treatment could reduce the new bone formation of AS.

## Introduction

Ankylosing spondylitis (AS) is a sort of inflammatory arthritis that affects axial skeleton, peripheral joints, and certain extra-articular organs, including the eyes, skin, and gut (1–3). The occurrence of AS is insidious and progressive, leading to spinal deformity, loss of ability to work, disability, and quality-of-life deterioration (4). Although the prevalence of AS varies greatly across geographic regions (5), it has increased from 0.04 to 0.09% in the USA in recent decades (6). Several researchers have explained this increasing tendency partly by hygiene hypothesis (7), supporting an inverse relationship between worm infection and T helper type 1/17 (Th1/17)-based inflammatory disorders such as rheumatoid arthritis, inflammatory bowel disease, type 1 diabetes, and multiple sclerosis. Although the exact mechanism of AS remains largely unclear, Th1/17 pro-inflammatory mediators are strongly implicated in the initiation and progression of this disease (8–10). Treatment with Tumor necrosis factor (TNF) blockers improves physical function, disease activity, and health-related quality-of-life outcomes (11, 12), but not all AS patients respond to the medication. Besides, it is controversial currently whether TNF blockers prevent progression to ankylosis (13, 14). Thus, a novel therapeutic strategy for AS is needed.

Recently, many attempts have been made to use parasite administration (*e.g.*, ingestion of eggs of the nematode *Trichuris suis*) as a new modality for treating autoimmune disorders (15, 16). In animal models of rheumatoid arthritis, some helminth species or their proteins were able to reduce the severity of the clinical symptoms (17–22). However, the role of any trematodes-induced proteins in the pathogenesis and disease activity of AS has yet to be reported. Thus, this study aimed to assess the therapeutic potential of *Clonorchis sinensis*-induced proteins (CSp) for AS. Results of this study will provide a basis for further clinical applications of *C. sinensis.*

## Materials and Methods

### Human Samples

All patients satisfied the modified New York (NY) criteria for AS (23). Peripheral blood mononuclear cells (PBMCs) and synovial fluid mononuclear cells (SFMCs) were obtained from patients with active AS. The demographic characteristics of the patients are shown in **Table 1**. This study was carried out in compliance with the Helsinki Declaration. It was approved by the Ethics Committee. Written informed consent was obtained from all subjects (CNUH-2011-199).

**Table 1:** Clinical characteristics of ankylosing spondylitis.

	Peripheral blood mononuclear cells	Synovial fluid mononuclear cells
Total number	6	8
Age, mean ± SD (years)	24 ± 9.5	39.1 ± 13.7
Male, n (%)	6 (100.0)	4 (50.0)
BASDAI, mean ± SD	5.4 ± 4.0	7.6 ± 2.1
AS-DAS, mean ± SD	3.6 ± 2.1	
HLA-B27, n (%)	6 (100.0)	6 (75.0)
CRP, mean ± SD (range, mg/dl)	2.4 ± 1.6	4.6 ± 3.6
Recent treatments (last three months)		
Naive, n (%)	3 (50.0)	2 (25.0)
NSAIDs use, n (%)	3 (50.0)	5 (62.5)
Sulfasalazine use, n (%)	1 (16.6)	4 (50.0)
TNF-blocker use, n (%)	0 (0.0)	3 (37.5)

BASDAI, Bath Ankylosing Spondylitis Disease Activity Index; HLA, Human Leukocyte Antigen; CRP, C-reactive Protein; NSAIDs, Non-steroid inflammatory drugs; TNF, Tumor Necrosis Factor.

### *Clonorchis sinensis* Crude Antigen Preparation

Frozen *C. sinensis* adult worms were mixed with 1 ml of homogenation buffer (5 mM EDTA, 1% NP-40, 0.2 mM PMSF), homogenized, vortexed for 5 min, and centrifuged at 13,000 rpm for 20 min at 4°C. After centrifugation, the supernatant was used for protein extraction using Pierce BCA Protein Assay Kit (Thermo Scientific Co., Rockford, IL, USA) according to the manufacturer’s guideline.

### Cell Viability Assay

To determine cell proliferation and cytotoxicity, cells were seeded and stimulated with different concentrations of CSp for indicated time durations. Cell viabilities of PBMCs and SFMCs according to CSp treatment were investigated using a Cell Titer 96 AQueous One Solution Reagent (G3580, Promega, USA). Briefly, 100 μl RPMI was mixed with MTS solution (20 μl/well) and added to each well. After incubation, absorbance was recorded at the wavelength of 490 nm with a 96-well microplate reader (Molecular Devices, USA). For each flow cytometry analysis, whole cells were surface stained with anti-Fixable Viability Dye-eFluor780 (65-0865-14, Invitrogen, USA).

### Co-Culture of Human Inflammatory Cells With CSp

PBMCs and SFMCs were isolated and suspended in a complete medium (RPMI 1640, 2 mM L-glutamine, 100 units/ml of penicillin, and 100 μg/ml of streptomycin) supplemented with 10% fetal bovine serum (FBS; Gibco BRL, Grand Island, NY, USA), and then seeded into 96-well plates at cell density of 1 × 10^6^ cells/well. Cells in a 96-well culture plate were treated with CSp and then were activated with Dynabeads Human T-Activator CD3/CD28 (11131D, Gibco, USA) to obtain a bead to cell ratio of 1:1. Cells were then incubated in a humidified CO_2_ incubator at 37°C for 24 h. After stimulating with PMA (100 ng/ml) and ionomycin (1 μM) for 4 h, cells were stained with Pacific Blue-conjugated anti-CD4 (300521, BioLegend, USA), and PE-conjugated anti-CD45RO (304205, BioLegend, USA). Cells were washed, fixed, permeabilized with Cytofix/Cytoperm buffer, and stained intracellularly with FITC-conjugated anti-IFN-**γ** (552887, BD, USA), APC-conjugated anti-IL-17A (512334, BioLegend, USA) antibodies followed by analysis with FlowJo Software (BD, USA). In *ex vivo* cultured supernatants from PBMCs, INF-**γ**, IL-17A, TNF-α, and IL-6 were measured using ELISA (88-7316, 88-7176, 88-7346, and 88-7066, Invitrogen, Austria). The OD was recorded by a SpectraMax^®^ M2(Molecular Devices Corp., USA) set at 450 nm.

### Experimental Animal Model and Clinical Score

SKG mice on a BALB/c background were purchased from Clea Japan (Tokyo, Japan) and bred under a specific pathogen-free facility. These mice were kept in individually ventilated cages and provided with water and standard diet *ad libitum*. All experiments were approved by the Institutional Animal Care and Use Committee (CNU IACUC-H-2018-35). They were conducted under the Laboratory Animals Welfare Act, Guide for the Care and Use of Laboratory Animals. Female mice were used in this study. Experiments had three groups: negative control (*n* = 10 mice), vehicle group (VH, *n* = 10 mice), and CSp treatment group (*n* = 10 mice). For both VH and CSp treatment groups, a suspension of curdlan (Wako, Osaka, Japan) was intra-peritoneally (i.p.) administered at 3 mg/kg to mice aged 11 weeks. CSp treatment group received CSp (6 μg/0.2 ml) i.p. twice before arthritis induction. The same dosage was then maintained once a week until sacrifice. Control and VH groups received PBS i.p. instead of CSp. At the start of treatment, randomization was performed based on serial number generation. Clinical signs of mice were monitored twice a week and scored by two independent observers. Scores of the affected joints were summed as follows: 0 = asymptomatic, 1 = slightly swelling of the ankles or toes, 2 = ankle swelling severely, 3 = ankle severely swelling and toe swelling, and 4 = ankle and toe swelling and twisting). Sixteen points were the highest possible points.

### Positron Emission Tomography and Micro-Computed Tomography Analysis

A day before sacrifice, mice were fasted for 16 h prior to undergoing PET/micro CT. Briefly, mice were anesthetized followed by an i.v. injection of 18.5 MBq ^18^F-FDG and scanned sequentially, starting at 30 min post-injection using a small animal PET-CT (SEDECAL, SuperArgus PET/CT 4r, Spain) with a detachable animal bed for maintaining animal position. Anesthesia was maintained by inhalation of approximately 1.5% isoflurane/O_2_ for 1 L/min for individual scans and 2 L/min to obtain mouse hotel scans administered *via* nose cone. PET images were reconstructed using OSEM3D (ordered subset expectation maximization)/MAP (maximum a posteriori) algorithm. The volume of interest (VOI) with a diameter of 6-mm was drawn at both sides of the hind paws. Maximal and mean standardized uptake values (SUVx) were then measured. The following CT scan parameters were employed: energy/intensity of 40 kV, electric current of 500 μA, sample time of 40 ms, and resolution of 768 × 972 pixels. Before CT scan, QRM-MicroCT-HA phantom (QRM GmbH; Moehrendorg, Germany) was used for calibration. For segmentation of newly formed bone and normal mature bone, segmentation thresholds values of hind paws and caudal vertebrae were used as described in a previous study (24).

### Histological Findings

At the experimental endpoint, specimens of the ankle and gut were obtained from mice and fixed with 10% formalin for one week. After fixation, specimens were decalcified in 10% formic acid with shaking at 37°C for a week and embedded in paraffin. Paraffin blocks were sectioned at a thickness of 3.5 µm and deparaffinized in neo-clear (109843, Merck, USA), hydrated with graded ethanol, and stained with hematoxylin (105174, Merck, USA) and eosin (HT110216, Sigma, USA). All staining procedures followed standard protocols. Two blinded readers performed pathologic scoring for enthesitis (25) and enteritis (26).

### Other Methods

RNA sequencing, additional flow cytometry, immunoblotting procedure, and sodium dodecyl sulphate-polyacrylamide gel electrophoresis (SDS-PAGE) analysis are described in detail in **Supplementary Data 1**.

### Statistical Analysis

Symptom score data were assessed using two-way ANOVA with time as a dependent variable. Statistical significance of the difference between means was assessed using the Kruskal–Wallis test with Dunn’s multiple comparisons, Wilcoxon matched-pairs signed rank test or Mann–Whitney test. All statistical analyses were performed using Prism 5.0 Software (GraphPad Software, San Diego, CA, USA). For all graphs, *P* value less than 0.05 was considered as significant and marked as follows: *P = 0.05–0.01; **P = 0.01–0.001, and ***P < 0.001.

## Results

### Cell Viability of CSp Treatment

**Figure 1:**
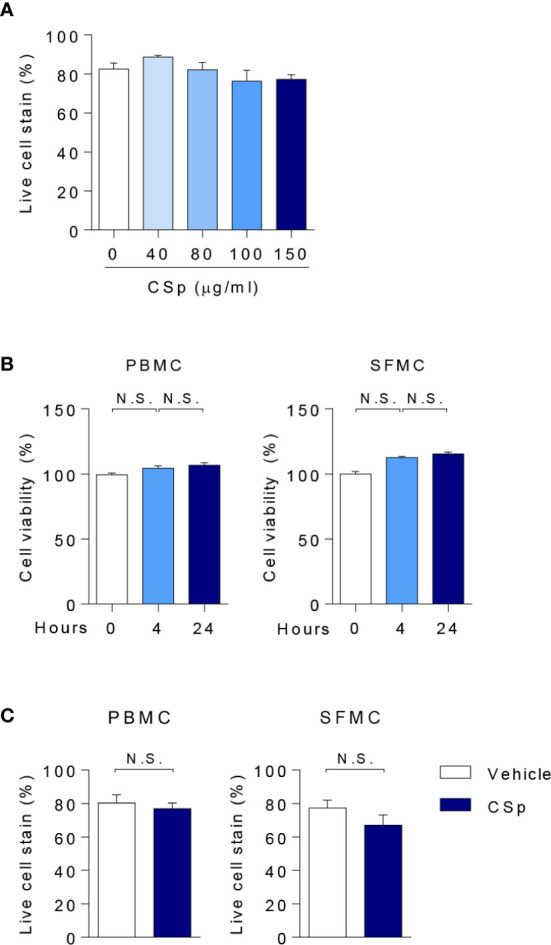
Assessment of cell viability with CSp. **(A)** Anti-Fixable Viability Dye of PBMCs were analyzed by flow cytometry depending on CSp concentration. Kruskal-Wallis test with Dunn’s multiple comparisons was performed to determine statistical significance. Values are the mean ± SEM. **(B)** Cell viability of PBMCs and SFMCs were analyzed by MTS assay depending on CSp treatment duration. Kruskal-Wallis test with Dunn’s multiple comparisons was performed to determine statistical significance. Values are the mean ± SEM. **(C)** Viability Dye of PBMCs and SFMCs were stained and measured. Mann-Whitney U test was performed to determine statistical significance. Values are the mean ± SEM. N.S: not significant. CSp: *Clonorchis sinensis*–derived protein; PBMCs: peripheral blood mononuclear cell; SFMCs: synovial fluid mononuclear cells.

There was no significant effect on cell viability with up to 150 µg/ml of CSp (**Figure 1A**). Treatment with CSp up to 24 h resulted in no reduced cell viability of PBMCs or SFMCs, analyzed by MTS (**Figure 1B**) and flow cytometry (**Figure 1C**). However, reduced viabilities were observed with CSp treatment for 48 and 72 h (**Supplementary Figure 1A**). In RNA sequencing analysis, down-regulation of several genes related to the metabolic pathway was found after CSp stimulation (**Supplementary Figures 1B, C**), but there was no significance in the genes related to the apoptosis (**Supplementary Figure 1D**). Expression levels of INF-**γ** and IL-17A in sequence data were also declined according to the presence of CSp (**Supplementary Figure 1E**).

### CSp Suppresses the Pro-Inflammatory Cytokines in *Ex Vivo* Experiment

**Figure 2:**
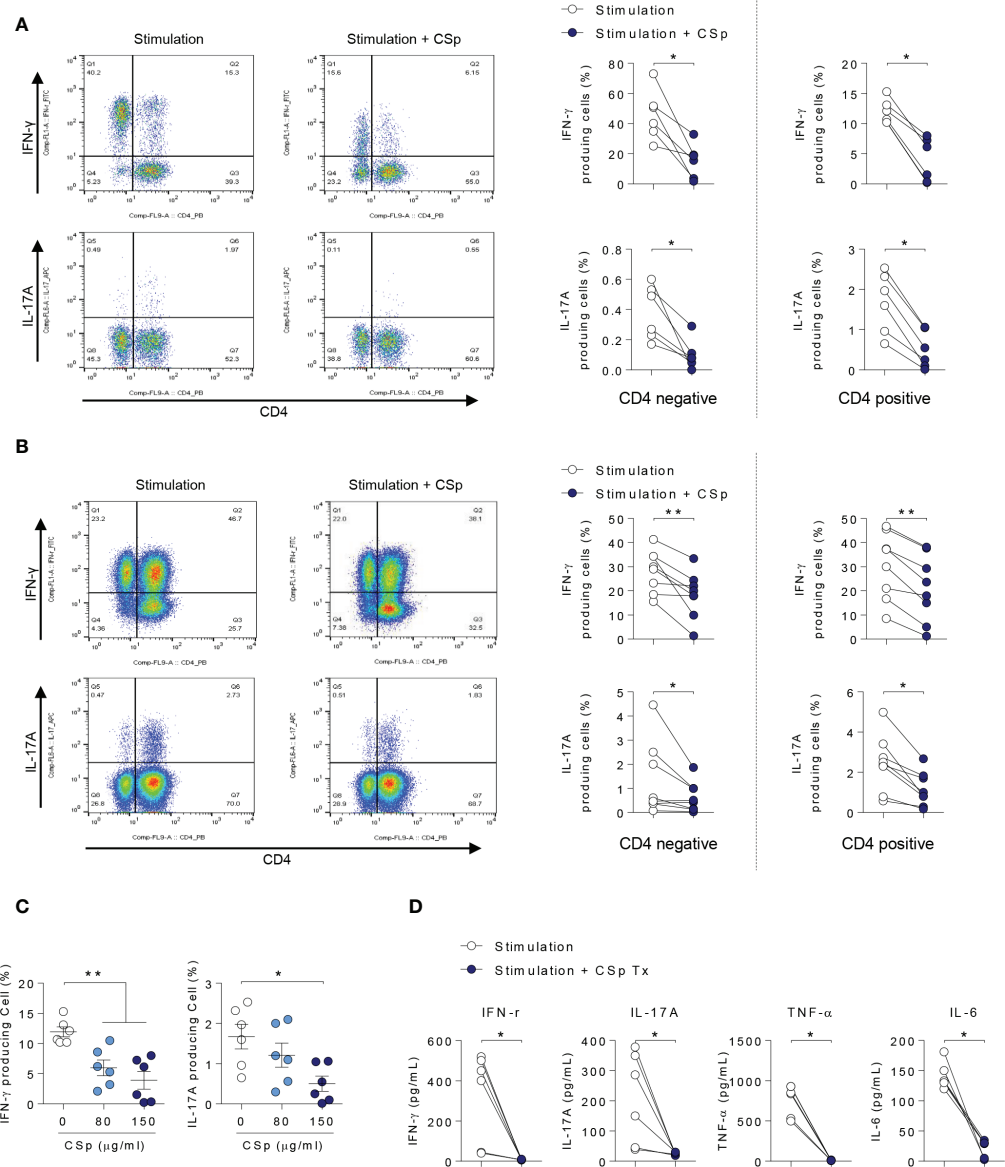
CSp treatment decreases the production of inflammatory cytokines from PBMCs and SFMCs in patients with ankylosing spondylitis. Cells were activated with Dynabeads human CD3/CD28 in the presence or absence of CSp for 24 hours. Then cells were stimulated with PMA and ionomycin for 4 hours. Percentages of INF-*γ* and IL-17A positive cells from PBMCs **(A)** and SFMC **(B)** were analyzed. **(C)** Percentages of INF-*γ* and IL-17A positive cells from PBMCs were analyzed depending on CSp concentration. Kruskal-Wallis test with Dunn’s multiple comparisons was performed to determine statistical significance. **(D)** In *ex vivo* cultured supernatants from PBMC, INF-*γ*, IL-17A, TNF-α, and IL-6 were measured by ELISA. Symbols represent the individual sample. *P < 0.05, **P < 0.01, by Wilcoxon matched-pairs signed rank test. CSp: *Clonorchis sinensis*–derived protein; PBMCs: peripheral blood mononuclear cell; SFMCs: synovial fluid mononuclear cells.

To explore the anti-inflammatory properties of CSp on cytokines in a human setting, PBMCs and SFMCs obtained from patients with active AS were stimulated and cultured *ex vivo* for 24 h in the presence or absence of CSp. In PBMCs and SFMCs, frequencies of IFN-**γ** and IL-17A producing cells in both memory CD4 negative and positive T cells were significantly reduced after treatment with CSp (**Figures 2A, B**; **Supplementary Figures 2A, B**). Dose dependent suppressive effects of IFN-**γ** and IL-17A production in PBMC were observed (**Figure 2C**). In the supernatant of PBMCs, the production of INF-**γ**, IL-17A, TNF-α, and IL-6 were significantly reduced after CSp treatment (**Figure 2D**). Additionally, further experiments were performed on the effect of CD8 positive T cells or Mucosal-associated invariant T (MAIT) cells in active AS patients. IFN-**γ** producing cells in the CD8 T cells were found to be significantly decreased after CSp treatment (**Supplementary Figure 2C**). Frequencies of IFN-**γ** and IL-17A producing cells in the MAIT cells were also found to be reduced after CSp treatment (**Supplementary Figure 2D**). To determine the role of CSp in inflammatory cell signaling, immunoblot assay was performed. We observed that the anti-inflammatory effect of CSp on immune cells was mainly due to the suppression of NF-kB phosphorylation (**Supplementary Figure 3**).

### CSp Treatment Suppresses Clinical Symptoms in SKG Mice

**Figure 3:**
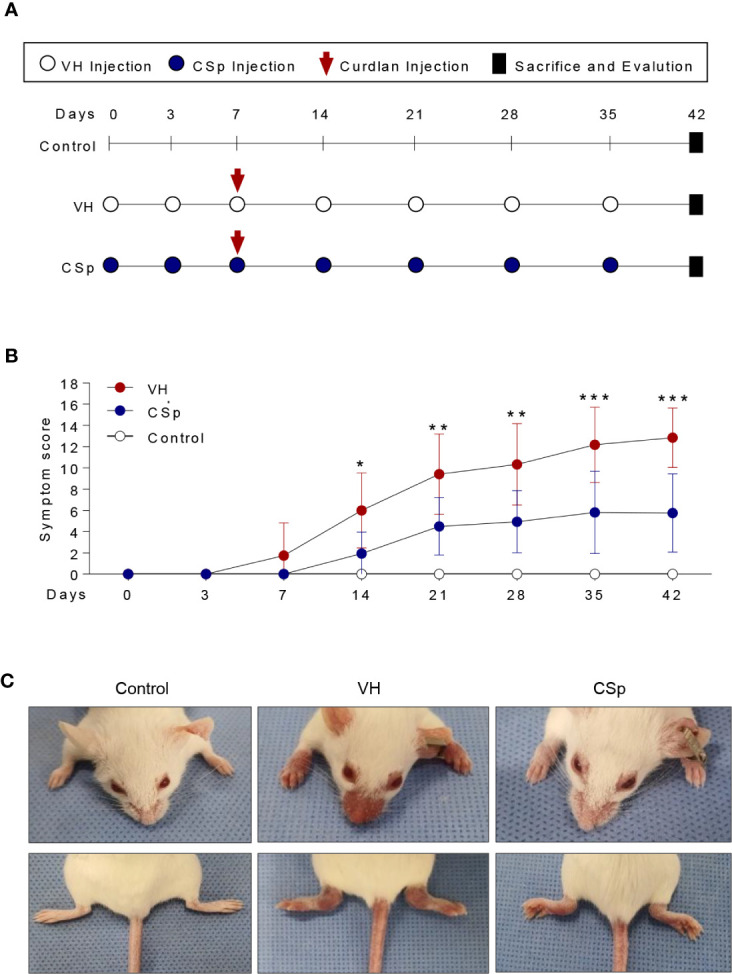
CSp treatment reduces clinical symptoms in SKG mice. **(A)** SKG mice were treated with CSp or vehicle i.p. for 6 weeks. **(B)** The arthritis scores were determined based on clinical arthritis severity in each group (n = 10 mice for each group). The values of the left panel are the mean ± SD. *P < 0.05, **P < 0.01, ***P < 0.001, by two-way analysis of variance (ANOVA). **(C)** Representative mouse from each group at the end of the experiment. CSp: *Clonorchis sinensis*-induced protein; VH: vehicle group.

**Figure 4:**
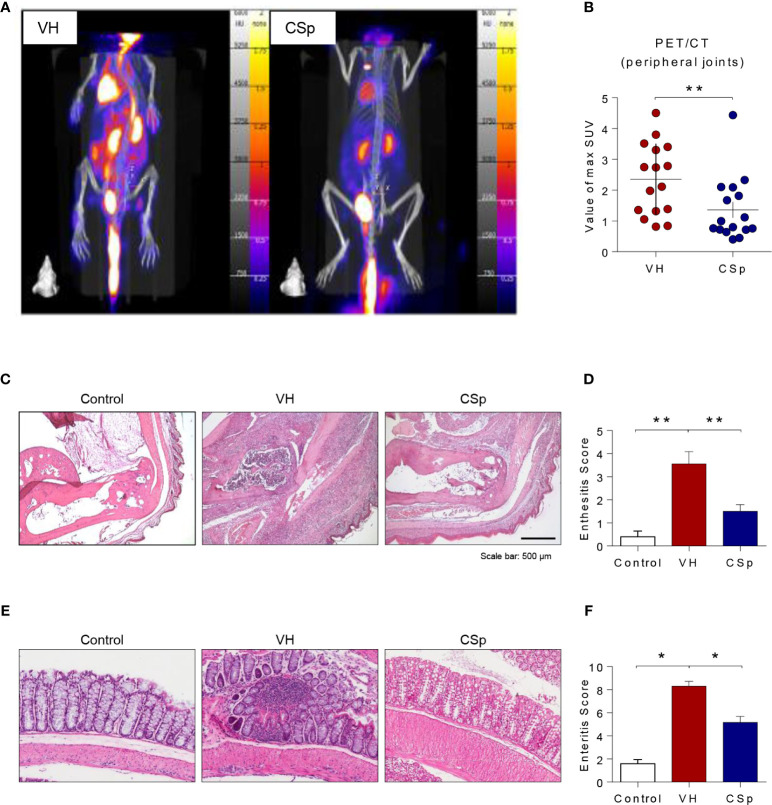
CSp treatment reduces arthritis, enthesitis, and enteritis in SKG mice. Mice were scanned using ^18^F‐FDG PET at the end of the experiment. **(A)** Representative PET images are shown in each group. **(B)** The mean values of max. SUV of the peripheral joint were measured. Symbols represent individual ankle joint (n = 8 mice for each group). **P < 0.01, was determined by Mann Whitney test. **(C)** Representative heel tissue stains at the end of the experiment are shown. **(D)** Analysis of histological scores for enthesitis was shown in bar graphs. **(E)** Representative gut tissue stains at the end of the experiment are shown. **(F)** Analysis of histological scores for enteritis was shown in bar graphs. Values are the mean ± SEM. *P < 0.05, **P < 0.01, by Kruskal-Wallis test with Dunn’s multiple comparisons. CSp: *Clonorchis sinensis*-induced protein; VH: vehicle group.

To investigate the effect of CSp on the development and progression of AS in curdlan-treated SKG mice, mice were treated with either CSp or VH from one week before curdlan injection (**Figure 3A**). The CSp treatment delayed the onset of arthritis and significantly reduced the severity of arthritis (**Figure 3B**). At the end of the experiment, CSp injection significantly suppressed the arthritis symptoms (5.7 ± 3.68 *vs.* 12.8 ± 2.78, *P* = 0.008). Although typical psoriatic skin lesions did not develop, VH-treated mice developed skin redness and hair loss around the ears and nose. However, skin lesions were not obvious in the CSp treatment group (**Figure 3C**). The frequency of IL-17A producing cells from SKG splenocytes in the CSp treatment group was significantly lower than that from VH-treated mice (2.74 ± 0.19 *vs.* 3.86 ± 0.39, *P* = 0.024). On the other hand, frequencies of IFN-*γ* and TNF-α producing cells were not significantly reduced in the CSp-treated group, compared to those from VH-treated mice (7.42 ± 0.64 *vs.* 10.35 ± 1.07, *P* = 0.114; 6.04 ± 1.43 *vs.* 13.75 ± 2.84, *P* = 0.087, respectively) (**Supplementary Figure 4**). A variety of targets for inflammation imaging have been discovered and utilized. Among them, ^18^F-FDG has been successfully applied in the inflammation realm. ^18^F-FDG-used PET features high sensitivity and specificity. PET has become one of the most frequently used molecular imaging techniques in the clinic (27). Therefore, we used PET images for measuring inflammation on peripheral joints. Representative images are shown in **Figure 4A**. Mean values of max. SUV of peripheral arthritis from the CSp-treated group were significantly lower than those from VH-treated mice (1.36 ± 1.03 *vs.* 2.35 ± 1.15, *P* = 0.0056) (**Figure 4B**). To assess the effect of CSp on local entheseal and gut inflammation, ankle and gut tissues were analyzed histologically. Representative tissue stains at the end of the experiment are shown in **Figures 4C, E**. Histologic assessment showed that mice treated with CSp had less enthesitis and enteritis than VH-treated mice (enthesitis score, 1.50 ± 0.57 *vs.* 3.55 ± 0.52, *P* = 0.0047; enteritis score, 5.17 ± 0.21 *vs.* 8.30 ± 0.43, *P* = 0.001) (**Figures 4D, F**), respectively.

### CSp Inhibits New Bone Formation in *In Vivo* Mice Model

**Figure 5:**
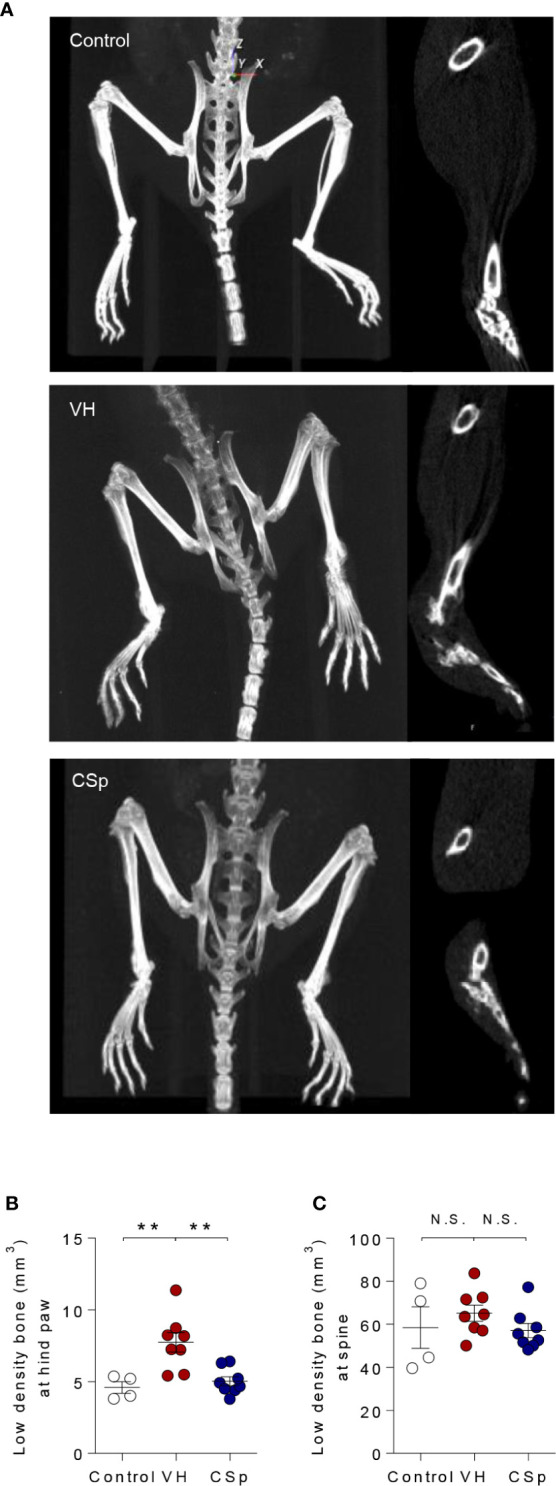
Reduction of new bone formation by CSp in SKG mice model. **(A)** Representative radiographic images are shown for each group. Quantification of low-density bone (used as a measure for new bone) in hind paws **(B)** and spine **(C)** from each group. Symbols represent individual mouse (n = 4 mice for control, n = 8 mice for VH and CSp group). N.S., not significant, **P < 0.01, by Kruskal-Wallis test with Dunn’s multiple comparisons. CSp: Clonorchis sinensis-induced protein; VH: vehicle group.

To evaluate whether CSp could reduce new bone formation, axial and peripheral joints were assessed by micro-CT at the end of treatment. Representative micro-CT images for each group are shown in **Figure 5A**. Analysis of hind paw revealed less low-density bone (used as a measure for new bone) in CSp-treated mice than in VH-treated mice (5.02 ± 0.92 *vs.* 7.73 ± 1.91, *P* = 0.001) (**Figure 5B**). CSp-treated mice exhibited spinal new bone formation similar to VH-treated mice (57.05 ± 9.40 *vs.* 65.15 ± 10.55, *P* = 0.3383) (**Figure 5C**). Quantification of normal-density bone (>800 mg of HA/cm^3^) revealed that mice with CSp treatment had similar bone volume to other groups, suggesting no reduction in normal bone loss (data not shown).

## Discussion

Helminth parasites inhabit immune-competent hosts for a long period of time and appear to develop strategies to induce strong anti-inflammatory responses in the infected host. The inverse prevalence between autoimmune diseases and helminth infection implies a potential protective role of helminth infection in autoimmune diseases. Yet, the effect of any trematode or their products on AS remains unclear. To the best of our knowledge, this is the first study that demonstrates the ameliorative effect of CSp on the clinical signs and cytokine derangements in AS. Moreover, the administration of this CSp could reduce new bone formation in an *in vivo* AS mice model.

Regarding the helminths therapy targeting arthritis, experimental infections with *S. japonicum, S. mansoni, A. suum*, and *H. diminuta*, or administration of their proteins have been shown to possess inhibitory effects on murine collagen-induced arthritis (18–22). In these studies, anti-arthritic activity is mediated by the up-regulation of the Foxp3^+^ Tregs with subsequent favorable modulation of both pro- and anti-inflammatory cytokines (21). We speculated that *C. sinensis*, a liver fluke found predominantly in Asia including Korea and China, could be an alternative candidate of *Schistosoma.* Our speculation was supported by previous studies showing an inhibitory effect of *C. sinensis* protein in mice models of asthma and enteritis by inducing CD4^+^CD25^+^Foxp3^+^ T cells or IL-10 secreting macrophages (28, 29).

Up to date, only one study has reported the effect of *C. sinensis* on arthritis (30). In that study, *C. sinensis-*direct infection has a bad effect on arthritis. It induced an abnormal immune response in mice with collagen-induced arthritis. Although preliminary results from human clinical trials have indicated that treating patients with inflammatory diseases using live helminth parasites has therapeutic potential, the use of helminth products as therapeutic agents might have advantages over a live infection. Thus, we assessed the effect of CSp treatment under suitable conditions excluding poor cell viability. Cell viability was reduced with prolong treatment of CSp. However, according to RNA sequence analysis, it might be related to down regulation of metabolic pathway rather than cell death signals maintaining suppression of IFN-**γ** and IL-17A (**Supplementary Figure 1**). Notably, this study showed a remarkable decrease of IFN-**γ** and IL-17A production after treatment with CSp in human PBMC and SFMCs, indicating inhibitory effect of CSp on both systemic circulation and regional site. This reduction of inflammatory cytokines was found in both CD4 positive and negative cells without significant changes in the cell proportions (**Supplementary Figure 5**). In addition, inflammatory cytokines were significantly decreased in CD8 T and MAIT cells after CSp treatment. We found that the anti-inflammatory effect of CSp on immune cells was mainly due to the suppression of NF-kB family of transcription factors, which was known to play essential roles in inflammation (31). This phenomenon might serve as the basis of the beneficial effect of CSp, thus provides protection against immune-mediated diseases. This finding leads us to clarify the evidence of therapeutic agents against AS using a murine model.

Using SKG mice model, we found that CSp treatment group presented markedly ameliorated disease-specific symptoms and a significantly decreased production of IL-17A in splenocytes. Levels of INF-**γ** and TNF-α seem to decrease in CSp-treated mice compared with PC mice, although the decrease was not statistically significant. PET imaging and histological findings supported the inhibitory effect of CSp treatment on arthritis, enthesitis, and enteritis. In line with our data, similar changes in cells and cytokines such as downregulation of pro-inflammatory cytokines (INF-**γ**, TNF-α, and IL-17A) have been found in animals infected with *S. mansoni* (18). Such certain common alterations in the immune response pattern due to parasite might have contributed to the reduced severity of Th1/17-mediated immune disorders including AS. Result of this study suggest that CSp treatment is able to attenuate the symptom severity of AS *via* systemic and local suppression of pro-inflammatory mediators, further suggesting the potential of therapeutic agents for treating AS.

It is noteworthy that our results suggest that CSp can suppress new bone formation in an *in vivo* mice model. Effective therapeutic approaches to AS remain a substantial clinical challenge as the suitability of TNF blockade for preventing new bone formation remains controversial. The IL-23/17 axis has been suggested to be a key player in AS pathogenesis and osteoblastogenesis directly (10, 32–34). In a recent study, a signal transducer and activator of transcription 3 (STAT3) phosphorylation inhibitor was demonstrated to suppress the new bone formation (35). Although the exact mechanism remains unknown, our data highlighted that CSp could be active to inhibit new bone formation in AS which might be accompanied by attenuation of IL-17A *via* JAK2/STAT3 pathway. CSp did not show significant suppression of spinal new bone formation in this mouse experiment. The current study highlights the further need for elucidating the role of CSp in osteoblastogenesis. Previous studies have shown that it took up to 6 months for observing spinal bony changes in murine models-treated with agents (36, 37). Therefore, inadequate timing of anti-osteogenic effect by CSp might be a reason for not inhibiting spinal new bone formation. In contrast to peripheral joint, new bone formation on spines was hardly seen in the images of this study. Therefore, quantitative analysis based on bone density may be an inappropriate tool for measuring spinal bony progression. Because several models mimicking aspects of human spondyloarthritis have been introduced (38), further studies using spinal ankylosis animal models will provide sufficient evidence whether CSp could suppress progression of new bone formation in spine.

This study has several limitations. First, we could not clarify the exact mechanism in the suppression of inflammatory response. However, this might be mediated by the upregulation of regulatory cell populations such as CD4^+^CD25^+^Foxp3^+^ T cells or IL-10 secreting macrophages (21, 28, 29). The inverse correlation of IL-10 with Th17 cells has been addressed both in humans and an experimental murine model (18, 39). All the findings of these studies support the potential of using CSp to achieve a balance between Th17 cells and Treg cells by altering IL-10 levels as a promising treatment for AS. Second, we have used CSp as crude extracts in the current study. Analysis of CSp by SDS-PAGE revealed many protein bands ranging from 10 to 70 kDa of relative molecular mass (**Supplementary Figure 6**). Further characterization of the CSp at a molecular level will increase our knowledge of host-parasite interaction that can be applied to standardized therapeutic modality. Third, CSp was administered preventively in the current *in vivo* study. Therapeutic effect of CSp in the late course of disease should be further assessed on established arthritis.

In summary, our results confirmed that CSp treatment could effectively ameliorate not only inflammation but also new bone formation in AS. Our finding showed that CSp could suppress the pathology associated with AS without compromising the host’s ability to fight disease, suggesting that therapies based on the mode of action of CSp might provide novel therapeutic targets for treating AS.

## Data Availability Statement

The original contributions presented in the study are included in the article/**Supplementary Material**. Further inquiries can be directed to the corresponding authors.

## Ethics Statement

The studies involving human participants were reviewed and approved by the Ethics Committee of Chonnam National University Hospital (CNUH-2011-199). The patients/participants provided their written informed consent to participate in this study. The animal study was reviewed and approved by Institutional Animal Care and Use Committee (CNU IACUC-H-2018-35).

## Author Contributions

YL, M-JK, EW, and T-JK conceived the study, participated in study design, data analysis, and were responsible for writing and submission of the final manuscript. YL, M-JK, SJ, S-HJ, P-RP, KP, JK, and J-YK carried out the experimental studies. H-CS, SS, and T-HK analyzed and interpreted data. S-JH offered *C. sinensis* adult worms and interpreted data. HK analyzed RNA-sequence data. All authors contributed to the article and approved the submitted version.

## Funding

This study was supported by grants from the National Research Foundation of Korea (NRF) Grant funded by the Ministry of Education, Science, and Technology (grant no. NRF-2019R1C1C1004605; NRF-2019R1I1A3A01060016; NRF-2019M3E5D1A02067953; NRF-2017R1A2B4007994, NRF-2019R1A2C2004214), and by the Chonnam National University Hospital Biomedical Research Institute (grant no. BCRI 19047 and BCRI 19048).

## Conflict of Interest

The authors declare that the research was conducted in the absence of any commercial or financial relationships that could be construed as a potential conflict of interest.

## Supplementary Material

Supplementary Figure 1 Further assessment of cell viability with CSp using MTS assay and RNA sequencing. **(A)** Cell viability of PBMCs and SFMCs were analyzed by MTS assay depending on duration treated with 150 μg/mL of CSp. **(B, C)** Gene expression profiles related to the metabolic pathway after CSp treatment. **(D)** Gene expression profiles related to the apoptosis after CSp treatment. **(E)** Gene expressions of INF-*γ* and IL-17A by RNA sequencing were shown according to the presence of CSp. CSp: *Clonorchis sinensis*–derived protein; PBMC: peripheral blood mononuclear cell; SFMCs: synovial fluid mononuclear cell; VH: vehicle group.

Supplementary Figure 2 Flow cytometry gating strategies and other T cell response analysis. **(A, B)** The gating strategies for memory T cells were showen for PBMCs and SFMCs. **(C)** The production of IFN-γ by CD8 T cells among PBMCs were gated and analyzed after CSp stimulation. **(D)** Inflammatory cytokines from MAIT cells were stained analyzed after CSp stimulation. Symbols represent the individual sample. CSp: *Clonorchis sinensis*–derived protein; MAIT: Mucosal-associated invariant T; PBMCs: peripheral blood mononuclear cells; SFMCs: synovial fluid mononuclear cells.

Supplementary Figure 3 Immunoblot analysis of immune cells depending on CSp. Peripheral blood mononuclear cells with or without CSp were activated with PMA and ionomycin for 4 hours. These cells were then lysed for protein extraction and immunoblotting. Representative results of the immunoblot assay are shown.

Supplementary Figure 4 CSp inhibits IL-17 production on splenocytes in SKG mice. Splenocytes were isolated and stimulated with PMA and ionomycin for 4 hours. Percentages of INF-*γ*, IL-17A and TNF-α positive cells from total lymphocytes were gated **(A)** and analyzed **(B)**. Symbols represent the individual sample. **P* < 0.05, by Mann Whitney test. VH: vehicle; CSp: Clonorchis sinensis–derived protein.

Supplementary Figure 5 CSp does not affect on the proportion of lymphocytes among the peripheral blood mononuclear cells **(A)** and synovial fluid mononuclear cells **(B)**.

Supplementary Figure 6 Sodium dodecyl sulphate–polyacrylamide gel electrophoresis (SDS-PAGE) analysis of soluble proteins extracted from *C. sinensis*. SDS-PAGE showed many protein bands ranging from 10 to 70 kDa of relative molecular mass. Among them, 10-kDa protein was the main molecule in *C. sinensis* crude extracts.

## Abbreviations

AS, Ankylosing spondylitis; CSp, *Clonorchis sinensis*-derived protein; i.p., intra-peritoneally; CT, computed tomography; NC, negative control; PBMC, peripheral blood mononuclear cell; PC, positive control; PET, Positron emission tomography; SFMC, synovial fluid mononuclear cells; TNF, Tumor necrosis factor.
