# Supplementary figures and images for: Clonorchis sinensis-Derived Protein Attenuates Inflammation and New Bone Formation in Ankylosing Spondylitis
Source: Front Immunol. 2021 Feb 25;12:615369. doi: 10.3389/fimmu.2021.615369 (PMC7947613; doi:10.3389/fimmu.2021.615369)

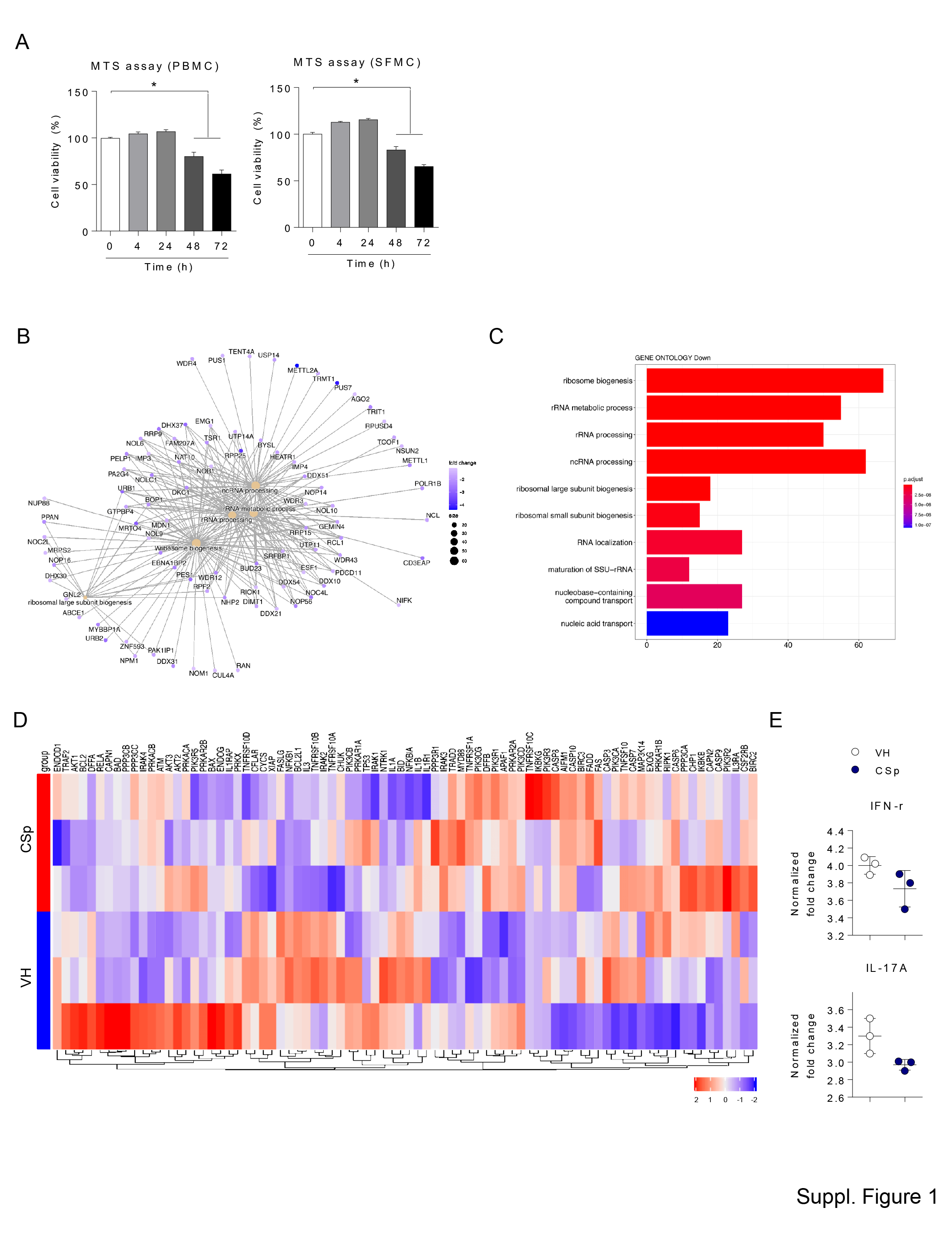

Supplement: Supplementary Figure 1 — Further assessment of cell viability with CSp using MTS assay and RNA sequencing. (A) Cell viability of PBMCs and SFMCs were analyzed by MTS assay depending on duration treated with 150 μg/mL of CSp. (B, C) Gene expression profiles related to the metabolic pathway after CSp treatment. (D) Gene expression profiles related to the apoptosis after CSp treatment. (E) Gene expressions of INF-γ and IL-17A by RNA sequencing were shown according to the presence of CSp. CSp: Clonorchis sinensis–derived protein; PBMC: peripheral blood mononuclear cell; SFMCs: synovial fluid mononuclear cell; VH: vehicle group. [file Image_1.tif]

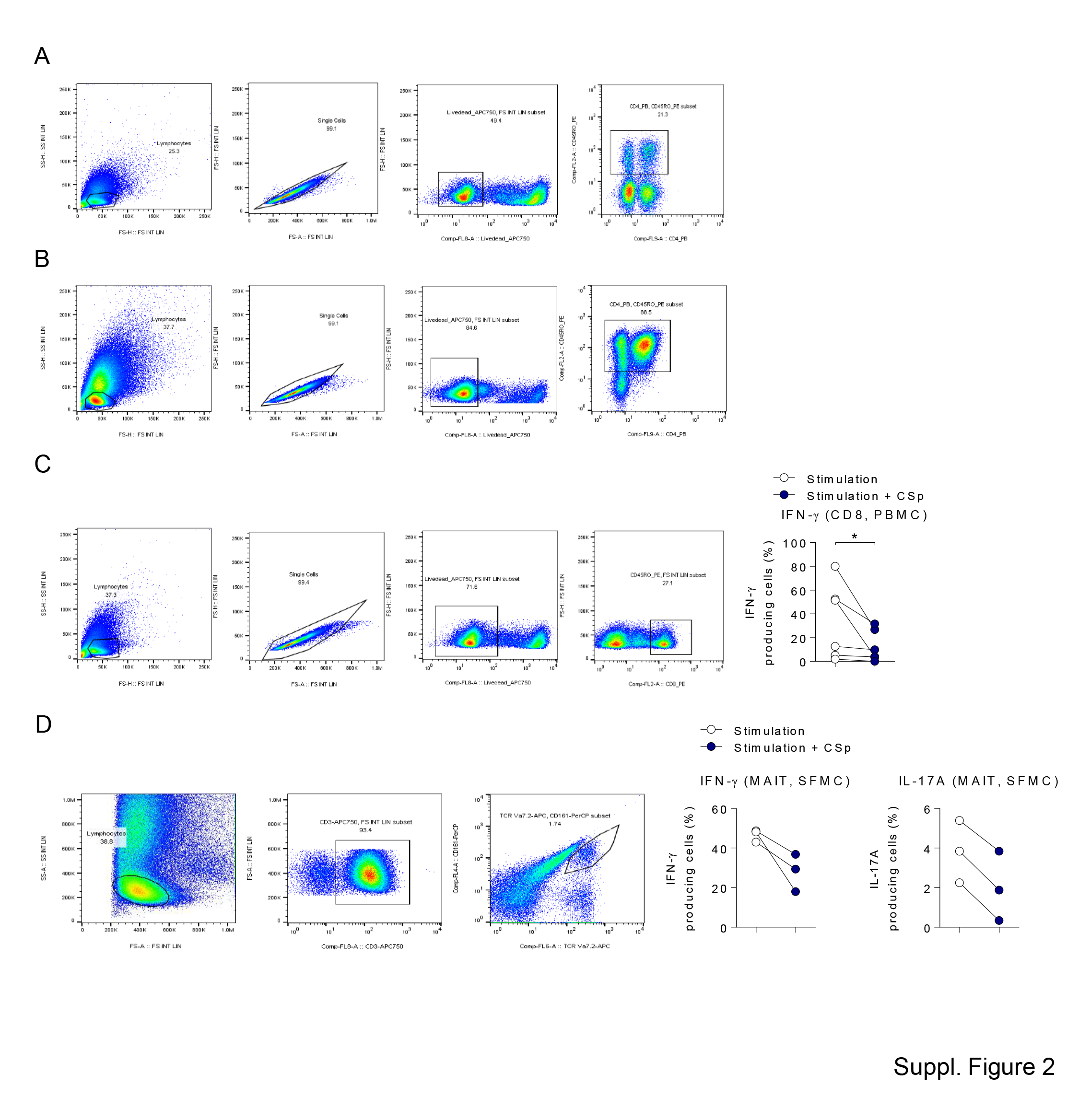

Supplement: Supplementary Figure 2 — Flow cytometry gating strategies and other T cell response analysis. (A, B) The gating strategies for memory T cells were showen for PBMCs and SFMCs. (C) The production of IFN-γ by CD8 T cells among PBMCs were gated and analyzed after CSp stimulation. (D) Inflammatory cytokines from MAIT cells were stained analyzed after CSp stimulation. Symbols represent the individual sample. CSp: Clonorchis sinensis–derived protein; MAIT: Mucosal-associated invariant T; PBMCs: peripheral blood mononuclear cells; SFMCs: synovial fluid mononuclear cells. [file Image_2.tif]

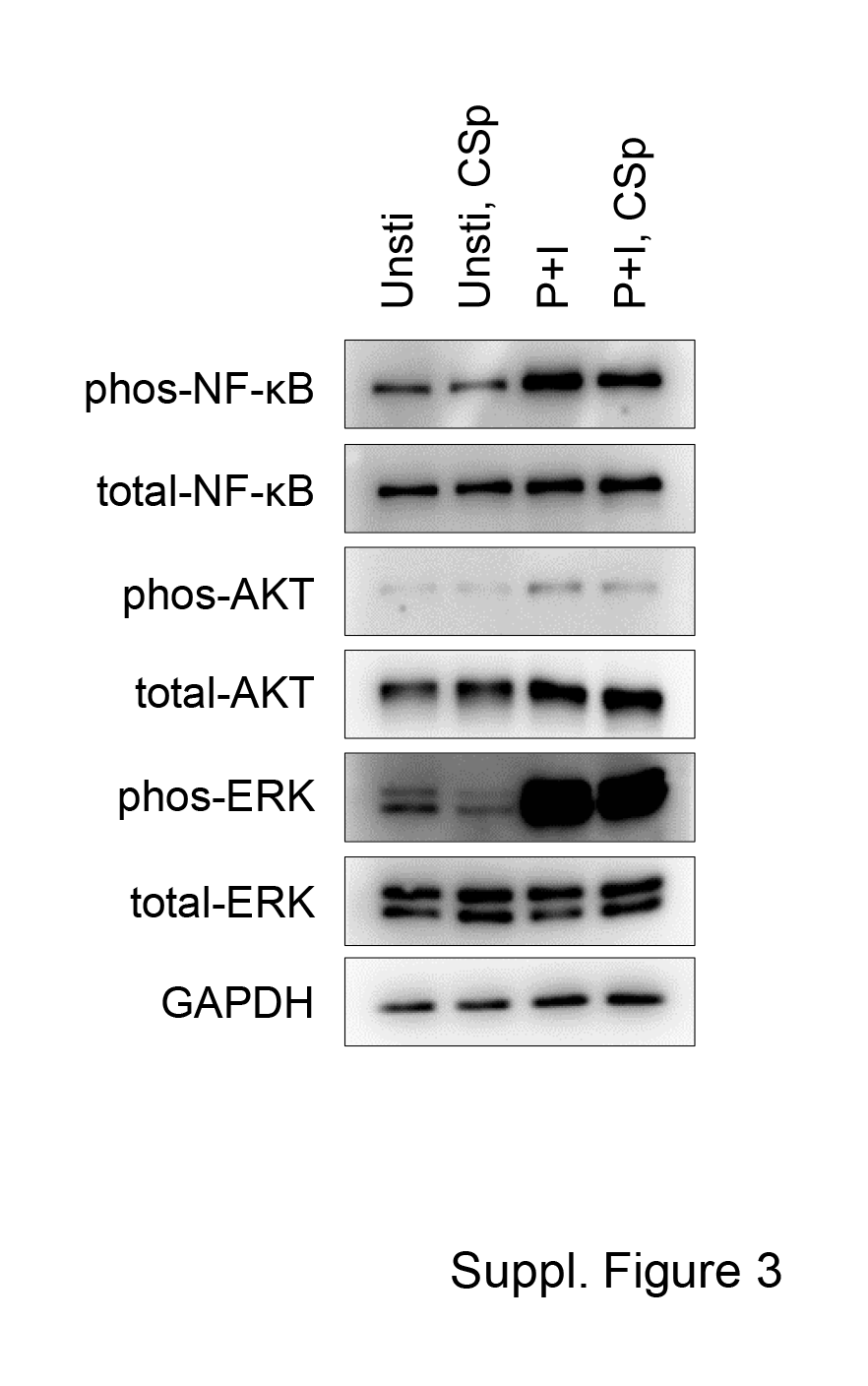

Supplement: Supplementary Figure 3 — Immunoblot analysis of immune cells depending on CSp. Peripheral blood mononuclear cells with or without CSp were activated with PMA and ionomycin for 4 hours. These cells were then lysed for protein extraction and immunoblotting. Representative results of the immunoblot assay are shown. [file Image_3.tif]

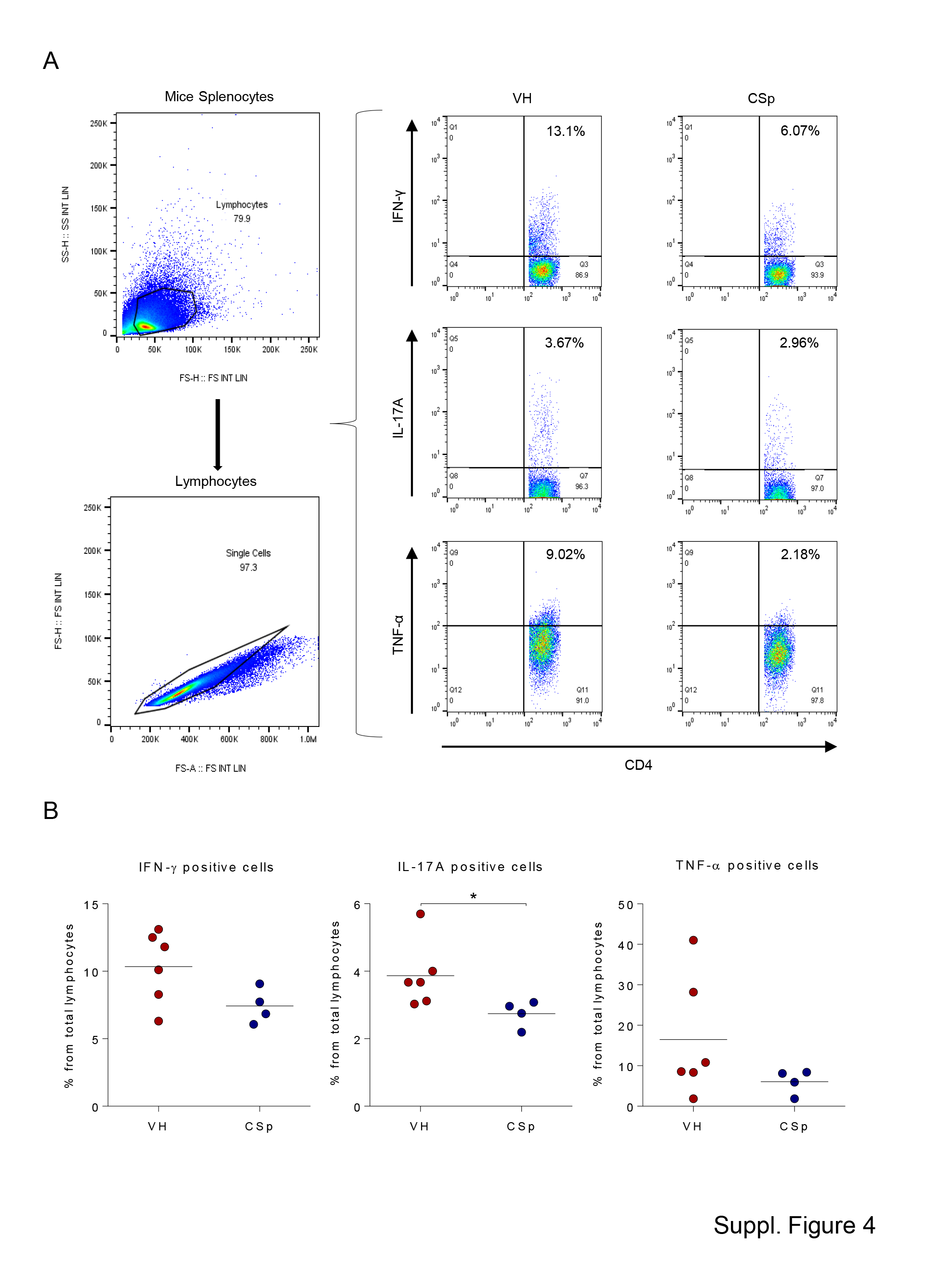

Supplement: Supplementary Figure 4 — CSp inhibits IL-17 production on splenocytes in SKG mice. Splenocytes were isolated and stimulated with PMA and ionomycin for 4 hours. Percentages of INF-γ, IL-17A and TNF-α positive cells from total lymphocytes were gated (A) and analyzed (B). Symbols represent the individual sample. *P < 0.05, by Mann Whitney test. VH: vehicle; CSp: Clonorchis sinensis–derived protein. [file Image_4.tif]

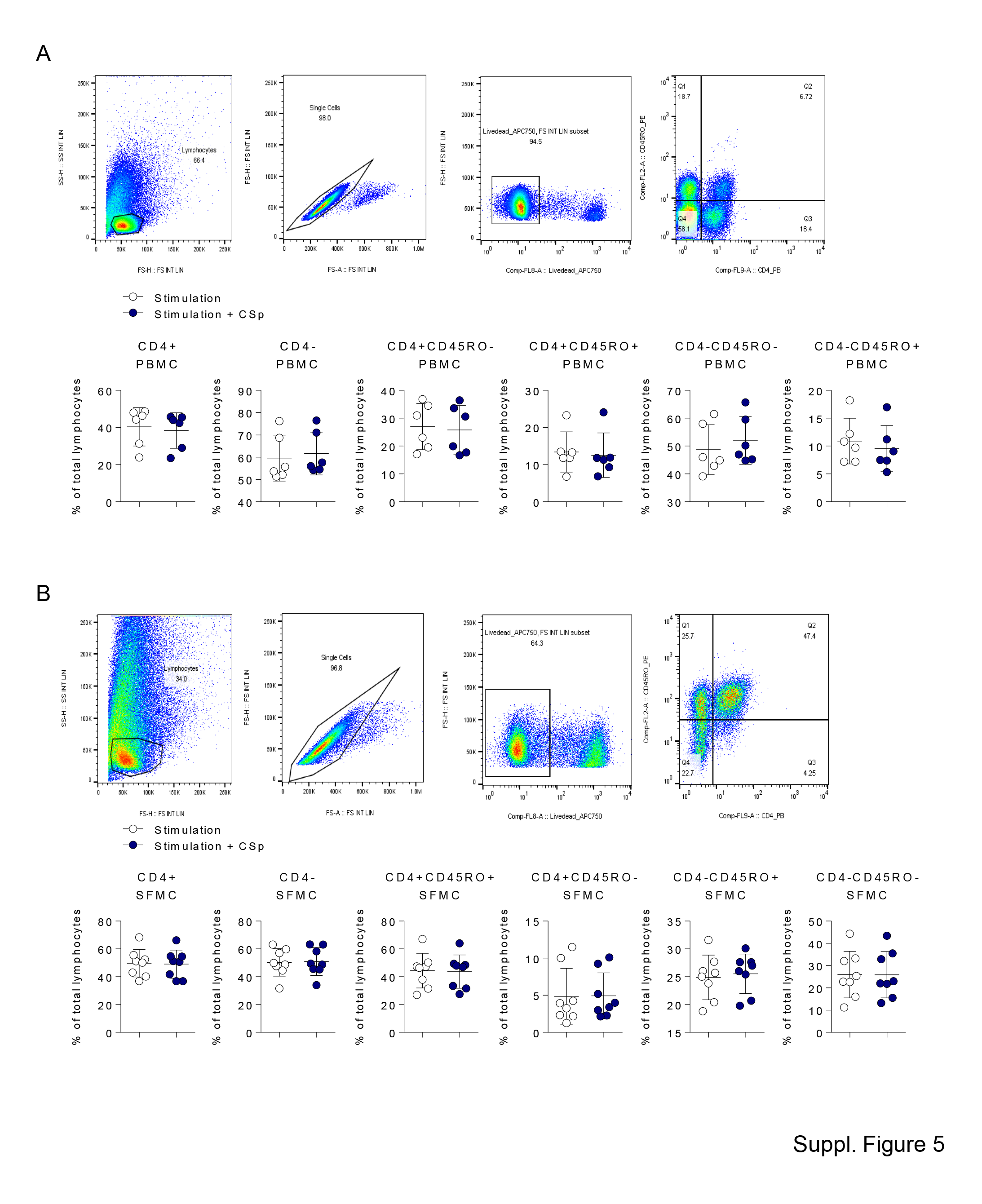

Supplement: Supplementary Figure 5 — CSp does not affect on the proportion of lymphocytes among the peripheral blood mononuclear cells (A) and synovial fluid mononuclear cells (B). [file Image_5.tif]

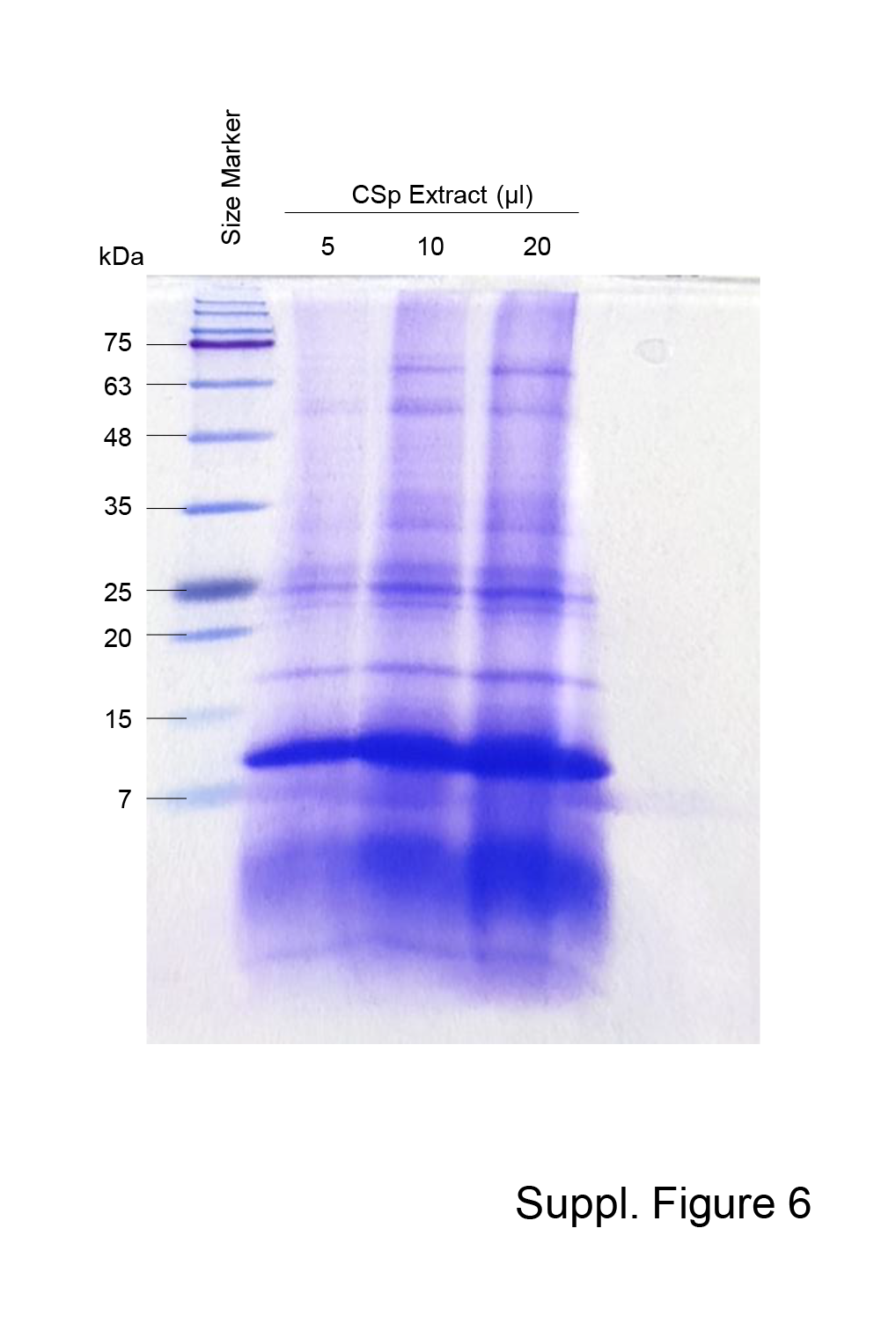

Supplement: Supplementary Figure 6 — Sodium dodecyl sulphate–polyacrylamide gel electrophoresis (SDS-PAGE) analysis of soluble proteins extracted from C. sinensis. SDS-PAGE showed many protein bands ranging from 10 to 70 kDa of relative molecular mass. Among them, 10-kDa protein was the main molecule in C. sinensis crude extracts. [file Image_6.tif]
