# Supplementary material for: Clonorchis sinensis-Derived Protein Attenuates Inflammation and New Bone Formation in Ankylosing Spondylitis
Source: Front Immunol. 2021 Feb 25;12:615369. doi: 10.3389/fimmu.2021.615369 (PMC7947613; doi:10.3389/fimmu.2021.615369)
Supplement: Supplementary file 7 [file DataSheet_1.docx]

Supplementary Material

**1. Supplementary materials and methods**

**Additional cell viability assay**

To determine cell proliferation and cytotoxicity with prolong incubation, cells were seeded and stimulated with CSp for 48-hours, and 72-hours. Cell viabilities of PBMCs and SFMCs according to CSp treatment were investigated using a Cell Titer 96 AQueous One Solution Reagent (G3580, Promega, USA). Briefly, 100 μl RPMI was mixed with MTS solution (20 μl/well) and added to each well. After incubation, absorbance was recorded at the wavelength of 490 nm with a 96-well microplate reader (Molecular Devices, USA).

**RNA-seq Data processing**

CD3/CD28 activated PBMCs were obtained from three healthy controls and were co-cultured with or without CSp for 24-h. Reads obtained from RNA sequencing were mapped against the UCSC hg38 reference file and further utilized hg38 gene transfer format (gtf) annotation format using STAR (version 2.6.0c) under default two pass (1). The resulting BAM files were used to

quantify count read per gene with HTSeq (version 0.10.0)(2) . All the count data are normalized with DESeq2 software package (version 1.24.0) to normalize the row counts into Variance-Stabilizing-Transformation (VST)(3).

**Differential gene expression, functional, and pathway enrichment analysis**

Differential gene expression analysis between control vs. experimental samples was determined by the significance criteria (adjusted *P* <0.05, |Log 2 (fold change) |>1. Gene ontology and pathway enrichment analysis were performed using cluster Profiler software package (version 3.12.0) (4) .

**Co-culture of Mucosal-associated invariant T (MAIT) cells or** **CD8 T cells with CSp**

PBMCs and SFMCs were isolated and suspended in a complete medium (RPMI 1640, 2 mM L-glutamine, 100 units/ml of penicillin, and 100 μg/mL of streptomycin) supplemented with 10% fetal bovine serum (FBS; Gibco BRL, Grand Island, NY, USA), and then seeded into 96-well plates at cell density of 1 × 10^6^ cells/well. Cells in a 96-well culture plate were treated with CSp and then were activated with Dynabeads Human T-Activator CD3/CD28 (11163D, Invitrogen, USA) to obtain a bead to cell ratio of 1:1. Cells were then incubated in a humidified CO_2_ incubator at 37℃ for 24 hours and then were stimulated by PMA (100 ng/mL) and ionomycin (1 μM) for 4 hours. For Mucosal-associated invariant T cells, cells were stained with APC750-conjugated anti-CD3 (555342, BD, USA), APC-conjugated anti-TCR Vα7.2 (351708, bioLegend, USA) and PerCP-conjugated anti CD161 mAbs (551138, BD, USA), FITC-conjugated anti-IFN-r (552887, BD, USA), PE-conjugated anti-IL-17A (560436, BD, USA), and PE-Cy7-conjugated anti-TNF-a (557647, BD, USA) antibodies. For CD8 T cell analysis, cells were stained with anti-Fixable Viability Dye-eFluor780 (65-0865-14, Invitrogen, USA) and PE-conjugated anti- CD8 (304205, biolegend, USA) antibodies. Cells were washed, fixed, permeabilized with Cytofix/Cytoperm buffer, and stained intracellularly with FITC-conjugated anti–IFN-γ (552887, BD, USA) antibody. Data were analyzed using FlowJo Software (BD, USA).

**Immunoblot**

Cells were lysed with 1X RIPA buffer containing phosphatase (5870S, Cells signaling, USA) and protease (535140, Calbiochem, USA) inhibitors. Proteins were quantified with a Bradford assay. Protein (10-30 μg) were subjected to immunoblotting. Antibodies used for immunoblotting were as follows: phos-NF-kB p65 (3033), phos-AKT (Ser473) (4060), total-AKT (4691), phos-ERK (9101), total-ERK (9102), and GAPDH (2118) from Cell Signaling Technology (Danvers, MA, USA). Total NF-kB p65 (sc-372) antibody was purchased from Santa Cruz Biotechnology (Dallas, TX, USA). Secondary antibody for goat anti-rabbit IgG (111-035-003) and goat anti-mouse IgG (115-035-003) were obtained from Jackson ImmunoResearch (West Grove, PA, USA).

**Mice splenic cell analysis**

The mouse spleen was isolated aseptically, and then gently ground in the RPMI1640 medium. These cells were harvested after mashing through a 40μm cell strainer and suspended in RPMI1640 supplemented with 10 % bovine fetal serum, 2 mM L-glutamine, 100 units/mL penicillin, and 100 mg/mL streptomycin. Lymphocytes from spleens were suspended in RPMI1640 and adjusted to have proper cell densities. After direct stimulating with PMA (100 ng/mL) and ionomycin (1 μM) for 4 hours. cells were then subjected to FITC-labeled anti- IFN-γ (505806, Biolegend, USA), followed by PE-labeled anti- IL-17 (506904, bioLegend, USA), APC-labeled anti-CD4 (100412, bioLegend, USA) and APC/cy7-labeled anti-TNF-α (506344, bioLegend, USA) for intracellular cytokine staining after using a Cytofix/Cytoperm kit (BD Biosciences) according to the manufactures’ instructions.

**SDS-PAGE analysis of CSp**

Soluble proteins extracted from *C. sinensis* were subjected to 12 % sodium dodecyl sulfate polyacrylamide gel electrophoresis (SDS-PAGE) as described by Laemmli (1970). The SDS-PAGE antigen separation was done under reducing conditions in gradient polyacrylamide gel cast with 12 % separating and 6 % stacking gel mixture. The electrophoresis was carried out in Mini Protean II electrophoresis apparatus (Bio-Rad, Hercules, CA) at 90 V for about 3–4 hours. Gels were stained with 0.05 % Coomassie brilliant blue. The molecular weights of proteins were then determined by comparing their migration distance against that of a known molecular marker. Gels were then visualized under Bio Rad gel documentation system.

**References**

1. Dobin A, Davis CA, Schlesinger F, Drenkow J, Zaleski C, Jha S, et al. STAR: ultrafast universal RNA-seq aligner. *Bioinformatics* (2013) 29:15-21. doi 10.1093/bioinformatics/bts635
2. Anders S, Pyl PT, Huber W. HTSeq--a Python framework to work with high-throughput sequencing data. *Bioinformatics* (2015) 31:166-9. doi 10.1093/bioinformatics/btu638
3. Love MI, Huber W, Anders S. Moderated estimation of fold change and dispersion for RNA-seq data with DESeq2. *Genome Biol* (2014) 15:550. doi 10.1186/s13059-014-0550-8
4. Yu G, Wang LG, Han Y, He QY. clusterProfiler: an R package for comparing biological themes among gene clusters. *OMICS* (2012) 16:284-7. doi 10.1089/omi.2011.0118
